# Learning stochastic finite-state transducer to predict individual patient outcomes

**DOI:** 10.1007/s12553-016-0146-2

**Published:** 2016-10-17

**Authors:** Patricia Ordoñez, Nelson Schwarz, Adnel Figueroa-Jiménez, Leonardo A. Garcia-Lebron, Abiel Roche-Lima

**Affiliations:** 1 Rio Piedras Campus, University of Puerto Rico, San Juan, Puerto Rico; 2 Interamericana University in Fajardo, Fajardo, Puerto Rico; 3 University College of San Juan, San Juan, Puerto Rico; 4 Medical Sciences Campus, University of Puerto Rico, San Juan, Puerto Rico

**Keywords:** Prediction of patient outcomes, Machine learning, Classification and visualization of physiological data

## Abstract

The high frequency data in intensive care unit is flashed on a screen for a few seconds and never used again. However, this data can be used by machine learning and data mining techniques to predict patient outcomes. Learning finite-state transducers (FSTs) have been widely used in problems where sequences need to be manipulated and insertions, deletions and substitutions need to be modeled. In this paper, we learned the edit distance costs of a symbolic univariate time series representation through a stochastic finite-state transducer to predict patient outcomes in intensive care units. The Nearest-Neighbor method with these learned costs was used to classify the patient status within an hour after 10 h of data. Several experiments were developed to estimate the parameters that better fit the model regarding the prediction metrics. Our best results are compared with published works, where most of the metrics (i.e., Accuracy, Precision and F-measure) were improved.

## Introduction

Current methods for measuring the well-being of a patient in the intensive care unit (ICU) acquire a patient’s vital signs data at rates that are difficult for a human to analyze (60–500 Hz). These measurements are displayed on a monitor for a few seconds as a collection of univariate time series and then lost to further analysis. Instead, a lower-frequency version of this data is stored in an electronic health record after validation by a medical provider at the rate of once every 15 min to once every several hours. Physicians make life-saving decision based on this lower-frequency data. Recently, however, there has been interest in storing and analyzing the high-frequency data using automated and semi-automated methods [1]. Many are recognizing the importance of analyzing this data as a multivariate temporal representation by creating multivariate probabilistic models [2] or temporal abstractions [3] from electronic health records or creating multivariate structures that are similar to those in other domains such as convolutional neural networks [4] or imaging [5].

In related work, four multivariate time series representations were examined to serve as compressed representation of high frequency physiological data from Intensive Care Units: Stacked Bags-of-Patterns, Multivariate Bags-of-Patterns, Multivariate Piecewise Dynamic Time Warping and Ensemble Voting with Bag-of-Patterns [6]. The representations were tested in three distinct data domains: field-motion capture data, robot sensor data, and ICU data. Two data sets were examined in each domain for a total of six different data sets. Similarity was measured by converting the data sets into the indicated representations and then classifying the data using the Nearest Neighbor algorithm. The results demonstrated that the multivariate representations outperformed univariate ones for the purpose of predicting the targeted outcome.

This paper represents work in the univariate time series domain which we are examining for application to the multivariate domain. We are using Stochastic Edit Distance on a concatenated symbolic representation of the time series to classify a physiological data set for an acute episode of hypotension. In this case, the edit cost probabilities are learned by a stochastic Finite-State Transducer (FST) [7]. We compare the best results in the multivariate domain to the results we achieved in the univariate domain with this new approach to test its potential effectiveness.

## Methods

### Dataset description

To compare results, we used one of the data sets from previous work [6] which came from the Physionet Challenge [8, 9]. It consists of 1–6 days of high-frequency physiological data from patients in an Intensive Care Unit (ICU). The prediction task was to classify which patients were going to enter an episode of acute hypotension in the forecast window of one hour after the last entry in the data set. We focused only on heart rate for this trail and instead of using leave one out validation, we used the test set that was provided in the Challenge and the original data from previous work as the training set.

The training set consisted of 58 of 60 patients from the 2009 Physionet Challenge, of which 28 experience an episode of hypotension in the hour following the period of the data sample. The data was taken from segments A and B which contained all the hours of data prior to the period in which the patient may or may not have experienced an episode of hypotension. Two patients were dropped because of large gaps in the training data resulting in the final size of 58. The test set consisted of segment B of Test Set A which contained 10 patients, of which half had experienced a period of acute hypotension following the sample data, and was composed of only 10 h of data.

### Data representation

The data for this paper was converted to a symbolic representation named Symbolic Aggregate approXimation (SAX) [10] which is explained in more detail in the Methods section. This representation normalizes the data in overlapping local windows prior to conversion such that the mean is 0 and standard deviation is 1. In some windows the variation in values was 0 which resulted in values that divided by zero. To correct for this, any subsequence that had zero variation was substituted by values of 0.

The second factor that required a difference in procedure for extracting the data was a matter of computational limitation. Our stochastic transducer, at the moment of this study, could not run on strings of length longer than 1020 characters. Consequently, the desired string lengths were achieved by using dimensionality reduction in the SAX algorithm. We reduced each string by counting any run of equal values as one value (i.e., abb abb abb aaa abb would be reduced to abb aaa aab, as abb is only counted once when repeated).

If this reduction was insufficient to achieve the desired length, all subsequences were joined into a single vector and we extracted the data using systemic random sampling. This sampling technique involves the selection of n elements from an ordered sampling frame of N elements, every k times. Systemic sampling was under the assumption that it best preserves any patterns along the time series data that was converted. A simple random sample may have, for example, involved more data from the earlier times of the time series, instead of equally across the sample.

If the single vector containing all the data has a length of 10,000 (*N*), and the desired length is 1000 (*n*), the k would be 10 (*N/n*). Every 10th element would be sampled. However, this assumes every single letter in our character string is independent from the rest. Instead, our SAX strings are grouped because they originate from time series subsequences. We sampled, not every k letter, but every k group of letters. The size of the group is determined by the size of alphabet parameter in the SAX algorithm. Following our previous example, if the vector is formed by groups of size 5 (i.e. using a, b, c, d, and e), our k would be every 50 ((N/n) * (size of alphabet) = 10 * 5). If every 50th element is sampled, the final sample would be 200 groups of 5 characters, hence a string of 1000 characters. Such sampling resulted in a decrease in the training set between 5 and 20 % of the original time series.

After the original vector of subsequences is reduced to a desired string length, it is concatenated to form a single string per time series. Each time series corresponds to a patient who either did or did not undergo an episode of hypotension. Patients who did undergo an episode of hypotension are classified as *1*, while other that did not is labeled *0*. Depending on the original time series classification, the final SAX string was attached to its appropriate label. The resulting output from our time series to SAX conversion was, for example, “*1 abbaccabcaba*...” or “*0 abaacbabcaaa*...”

### Algorithms

Before applying the learning Finite-Transducer for Stochastic Edit Distance to the time series data, the data was converted into the Symbolic Aggregate approXimation (SAX) representation [10]. This representation reduces the dimensionality of a time series by converting it into a collection of symbolic representations. The algorithm uses three variables: *WindowSize*, the number of values that can be represented by a SAX word; *Symbols*, the number of symbols in a SAX word (the bins on which the values are averaged for the representation); and finally *Alphabet*, the number of characters in a SAX word (the number of letters that can be used in the word to describe low to high values of distribution). Each SAX word represents the same amount of data and is calculated using an overlapping sliding window to capture all patterns in the data. These representations are a collection of words for each time series.

#### Definitions

Edit distance is widely used to compute similarities between pairs of strings. It is defined as the minimum number of operations, i.e. insertions, deletions, and substitutions, required to transform the input into the output. Stochastic edit distance is defined when the costs of the operations become random variables because the transformations are based on arbitrary phenomena. It can be modeled as a stochastic transduction, compiled in the form of a 2-tape automaton. This model is called stochastic finite-state transducer. It has resulted being very useful for sequence problems, such as pattern recognition, segmentation, DNA alignment and sequence classifications [7, 11, 12].

#### Learning stochastic edit distance

**Fig. 1:**
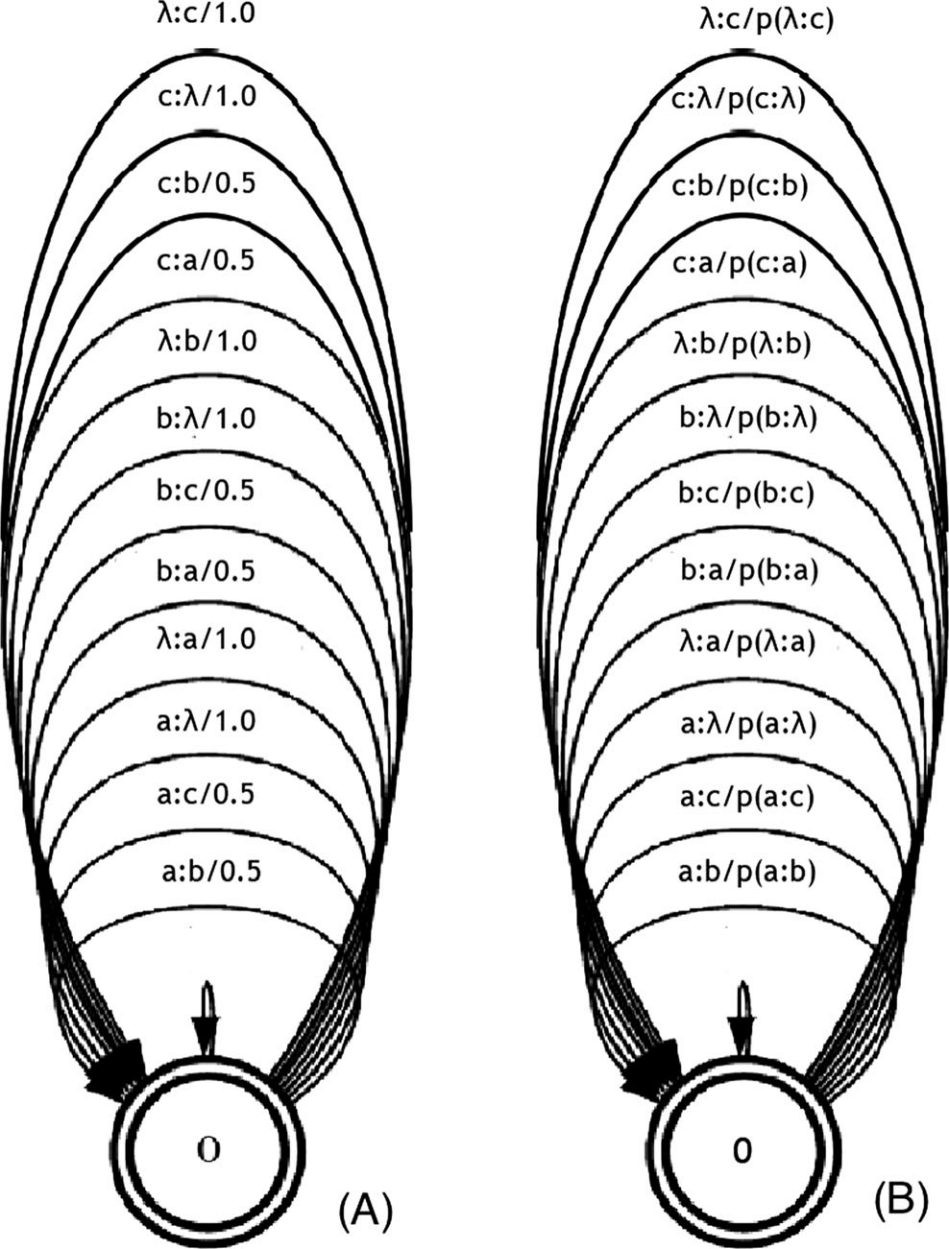
Finite-state transducer with alphabet [a; b; c; λ], where λ represent the empty symbol. **a** deterministic - with pre-define costs **b** stochastic - costs as probabilities

Figure 1a represents a finite-state transducer (FST), also called memoryless transducer, which allows one to compute edit distance using the pre-defined costs. Figure 1b represents a FST where the costs can be learned from a training set as the probability of each operation, i.e. Stochastic Finite State Transducers. In our case, an unbiased learning algorithm (*Algorithm 1*) using a stochastic conditional finite-state transducer was implemented to learn the probabilities associated with the edit distance costs.
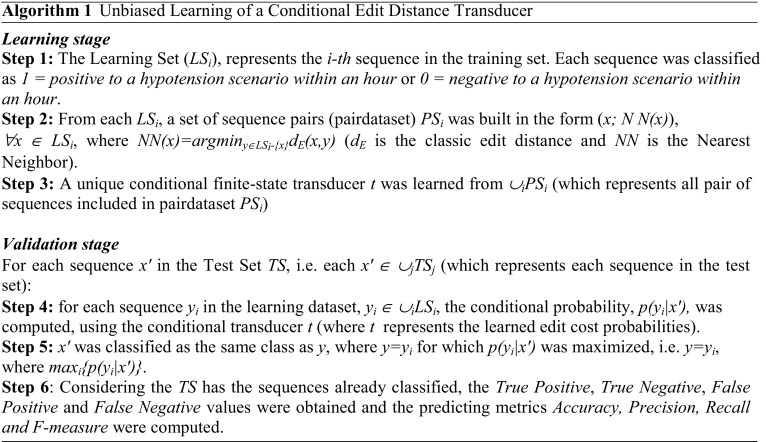

Algorithm 1 - ***Learning Stage*** used as training set the sequences from the 58 patients described in Section 2.1, which were obtained as a result of the time series conversions to SAX. **Step 1** created the learning set with the sequences and its classifications i.e. *1* and *0*.

**Step 2** created the pairdataset, *PS*, as the pair of sequences used as an input to learn the transducer. To create the pairdataset, for each sequence x the 1-Nearest Neighbor (*NN*) with the classic edit distance was computed, the pairs (*x,NN(x)*) were formed, both from the learning set.

The stochastic edit costs are obtained by learning the conditional finite-state transducer in **Step 3**. The recursive forward and backward algorithms were implemented to compute the probabilities *p(y|x)*, where *x*, *y* were the input data from the pairdataset [13]. Then, the expectation-maximization algorithm was used to optimize the parameters [14]. The expectation algorithm dealt with the problem of obtaining the matrix with the expected values, while maximization computed the current edit costs. These algorithms were repeated until the expected precision was reached (see [7] for more details about the algorithm).

In the ***Validation Stage***, the goal was to test the learned transducer with the edit cost probabilities on a real data set. This dataset consisted in *10* patients and included the sequences and their classifications (more details of the test set were also described in *Section* *2.1*). Then, for each sequence in the test set, the 1-Nearest-Neighbor was used along with the learned stochastic edit distances to classify the sequence as *0* or *1* based on the training set **Step 4 and 5**.

As the sequences in the test set were already classified, prediction metrics, i.e. Accuracy, Precision, Recall and F-measure, were computed to compare the results of the classifier under test with trusted external judgments. True Positive, True Negative, False Positive and False Negative values were used to compute the metrics, where the terms positive and negative refer to the classifier’s prediction and the terms true and false refer to whether that prediction corresponds to the external judgment (**Step 5**).

## Results

### Evaluation approach design

Several experiments are developed in order to obtain the most accurate model that evaluates univariate analysis on the high-frequency data. Implemented Learning and Validations Stages described in Algorithm 1 are repeated for different parameter values. These parameters were changed during the SAX conversion data. The idea behind these experiments is to establish the parameters that better adjust the stochastic finite-state transducer model to predict hypotension scenario within an hour.

These parameters, i.e. *WindowSize*, *Symbols* and *Alphabet*, are described in detail in Section 2.1. Nine different experiments are developed. In all cases, a *WindowSize* = 120 is used, because expert medical professionals felt that examining two hours of data was the minimal amount of data to capture a stark decline in a patient’s condition (an episode of acute hypotension). Parameters *Symbols* and *Alphabet* are changed by [6, 12, 24] and [3, 4, 5], respectively. The prediction metrics described in Algorithm 1 Step 5, i.e. Accuracy, Precision, Recall and F-measure values are then computed, using the following formulas:

$$ \begin{array}{l} Accuracy=\frac{truePos+ trueNegs}{\# instansces}\hfill \\ {} Precision=\frac{(truePos)}{truePos+ falsePos}\hfill \\ {} Recall=\frac{(truePos)}{truePos+ falseNegs}\hfill \\ {}F- Measure=\frac{2\ast Prec\ast Recall}{Prec.+ Recall}\hfill \end{array} $$

### Results by adjusted parameters

Table 1 describes the results of the experiments. As can be seen, parameters set for Exp. #1 and #2 are not even able to produce some of the validation metrics. That is because the data do not provide enough information to correctly learn the probability cost by the stochastic finite-state transducer. The model works better for larger Symbols parameter sizes (Exp. #7, #8, #9). The symbols parameter determines the granularity of the measurement meaning that in a *WindowSize* of 120 and *Symbols* value of 24, each symbol represents 5 min (120/24). The *Alphabet* value of 3 separates the normalized values into three symbolic representations based on whether the value falls into the low, average or high values in a Gaussian distribution. The best results are obtained when the parameters are set as *Symbols* = 24 and *Alphabet* = 3 (Exp. #7), where all the validation metrics are greater than 0.8 (i.e. Accuracy =0:85, Precision =0:82, Recall =0:87 and F-Measure =0:86).

**Table 1:** Results of the validation metrics by changing the parameters of the data conversion when stochastic finite-state transducer model is used to determine the stochastic edit distance costs

Exp.	Parameters			Results
#	Window Size	Symbols	Alphabet	
1	120	6	3	Accuracy =0.50
				Precision =0.0
				Recall = NaN
				F-Measure = NaN
2	120	6	4	Accuracy =0.5
				Precision =0.0
				Recall = NaN
				F-Measure = NaN
3	120	6	5	Accuracy =0.51
				Precision =0.23
				Recall =0.5
				F-Measure =0.31
4	120	12	3	Accuracy =0.76
				Precision =0.68
				Recall =0.78
				F-Measure =0.73
5	120	12	4	Accuracy =0.35
				Precision =0.28
				Recall =0.25
				F-Measure =0.26
6	120	12	5	Accuracy =0.68
				Precision =0.48
				Recall =0.69
				F-Measure =0.56
7	120	24	3	Accuracy =0.85
				Precision =0.82
				Recall =0.87
				F-Measure =0.85
8	120	24	4	Accuracy =0.69
				Precision =0.78
				Recall =0.69
				F-Measure =0.73
9	120	24	5	Accuracy =0.67
				Precision =0.81
				Recall =0.57
				F-Measure =0.67

### Discussion and related works

Based on the validation metrics in Exp. #7, our model makes a good prediction of the patient outcomes. Other results have been previously developed to predict patient status using time series data [6, 15]. Ordoñez et al. [6] use natural language processing and text mining techniques to predict patient outcomes. We use their study [6] as a baseline to compare with our method. They also compute the same prediction metrics (i.e. Accuracy, Precision, Recall and F-measure) to validate their results. Therefore, we compared the best values for each of the prediction metrics in their study and compared them with our results.

As can be seen in Table 2, our results are better than the previous for most of the metrics. Only the Recall metric gives better value when it is compared with our current research. It is also important to consider that we only use one univariate variable, Heart Rate, to predict hypotension while [6] uses a multivariate representation using Heart Rate and Mean Arterial Pressure.

**Table 2:** Metric Comparison: baseline vs current work

	Ordoñez et al. [5]	Current Work
Accuracy	0.81 ^a^	0.85
Precision	0.79 ^b^	0.82
Recall	0.96 ^c^	0.87
F-measure	0.84 ^d^	0.85

^a^Most accurate value from Multivariate Piecewise Dynamic Time Warping

^b^Most precise value from Multivariate Stacked Bags of Patterns

^c^Highest recall value from Multivariate Piecewise Dynamic Time Warping

^d^Highest F-measure value from Multivariate Piecewise Dynamic Time Warping

We had to reduce the size of the time series because of computational limitations to only 1020 characters. In [6], the multivariate representations with two variables outperformed the univariate representations. In this paper, even with more dimensionality reduction, the univariate representation outperformed the work of [6]. Although we have obtained good results, we still consider that Algorithm 1 implementation can be improved to handle larger sequences and multivariate representations to obtain more precise results.

## Conclusion and future works

In this research, we proposed a method to predict patient outcomes in Intensive Care Units which used probabilities of edit distance costs learned by stochastic finite-state transducer models. Time series data were converted to sequence representation to be used as a model input. Several experiments were developed by changing the parameters during the conversion process. We obtained good results based on the computed prediction metrics. When we compare with previous works, our proposal improved Accuracy, Precision and F-measure metric values.

In future work, other implementations of the algorithm in parallel will be used to increase the sequence length and improve efficiency. We would also like to create a multivariate representation of the algorithm. Additionally, other approaches using finite-state transducers can be used to improve prediction. For example, rational kernels (kernel based on finite-state transducers) can be combined with kernel methods, such as Support Vector Machine [15].
